# A School-Based Program to Promote Well-Being in Preadolescents: Results From a Cluster Quasi-Experimental Controlled Study

**DOI:** 10.1007/s10935-018-0530-y

**Published:** 2018-12-03

**Authors:** Elias Allara, Franca Beccaria, Roberta Molinar, Laura Marinaro, Antonella Ermacora, Alessandro Coppo, Fabrizio Faggiano, The Diario della Salute Evaluation Support Team, Alberto dal Molin, Angelo Di Dio, Simonetta Cossu, Rosita Reale, Simone Tonella, Milena Zoppi

**Affiliations:** 1 Department of Public Health and Primary Care, University of Cambridge, Cambridge, UK; 2 Department of Translational Medicine, Università del Piemonte Orientale, Via Solaroli 17, Novara, Italy; 3 Eclectica Institute of Research, Training and Communication, Turin, Italy; 4 Epidemiology Unit, Prevention Department, CN2 Local Health Authority, Alba, Italy

**Keywords:** School-based program, Well-being, Adolescence, Quasi-experimental study, Effectiveness, Health promotion, Prevention

## Abstract

*Diario della Salute [My Health Diary]* is a school-based program designed to enhance the subjective well-being and health of 12- to 13-year-old students. We hypothesized that providing students with the social and emotional skills to fulfill their potential and deal with common developmental tasks of adolescence (e.g., onset of puberty, identity development, increased responsibilities and academic demands) would result in improved well-being and health. The program comprises five standardized interactive lessons concerning common psychosocial and health issues in adolescence, and two narrative booklets addressed to both students and their parents. We evaluated the effectiveness of the program in terms of the students’ subjective well-being, aggressive behavior, and health behavior. Using a quasi-experimental study design, schools in the intervention group implemented the full program and those in the comparison group received their regular curriculum. We administered measures of the study’s objectives both before and after program implementation. Statistical analyses accounted for within-school clustering, potential socioeconomic and demographic confounding, and pre-implementation levels of these measures. We sampled 62 schools and allocated 2630 students to either an intervention or comparison group. Sociodemographic characteristics and baseline outcomes were balanced across study groups. Unexpectedly, respondents in the intervention group had 0.38 greater mean adjusted score of the WHO/Europe Health Behaviour in School-Aged Children Symptom Checklist instrument than respondents in the comparison group, indicating a reduction in subjective well-being. We did not observe any program effects on aggressive and health behaviors. The apparent reduction in subjective well-being reflected by an increased perception of psychosomatic complaints is suggestive of either increased emotional competence or, potentially, iatrogenic program effects. While greater emotional competence is positively associated with well-being over the course of life, the program in its present form should not be disseminated due to the possibility of adverse unintended effects.

## Introduction

In the last decade, there has been increasing interest in the potential of interventions capable of promoting psychological well-being among adolescents. Although most adolescents deal successfully with the developmental tasks that are typical for their age (e.g., onset of puberty, identity development, increased responsibilities and academic demands), some may not achieve optimal functioning and positive psychosocial adjustment. Failure to cope with the developmental tasks occurring in adolescence may impair future well-being, social functioning, and health status (Caprara, Barbaranelli, Pastorelli, Bandura, & Zimbardo, 2000; Yirmiya, 2007). Therefore, preadolescence represents an important opportunity for prevention and intervention.

The development of individual skills and social competence is not only useful in dealing with the developmental tasks of adolescence, but has also been found to be effective in promoting well-being and positive behaviors, and in protecting against emergent psychological disorders and problem behaviors (Cuijpers, van Straten, Smits, & Smit, 2006; Guerra & Bradshaw, 2008; Reddy, Newman, De Thomas, & Chun, 2009). Strategies designed to develop individual skills and social competence have been successfully employed in several prevention programs, such as the Paths curriculum (Kam, Greenberg, & Walls, 2003), the Gatehouse project (Bond et al., 2004), the Penn Resiliency Program (Gillham et al., 2007), Well-Being Therapy (Ruini et al., 2009), the Lars&Lisa Program (Possel, Baldus, Horn, Groen, & Hautzinger, 2005), Life Skills Training (Botvin & Griffin, 2004), and Unplugged (Faggiano et al., 2010). Most of these interventions have been implemented in schools, which constitute an ideal setting for addressing health and well-being in early adolescence. Compulsory school attendance facilitates universal prevention and the implementation of interactive group activities.

*Diario della Salute* [My Health Diary] is a school-based program designed to enhance subjective well-being and health among preadolescents by providing them with the social and emotional skills required to fulfill their potential and deal with the developmental tasks of adolescence. Compared to Unplugged which was the only evidence-based, school-based program available in Italy in 2012 when *Diario della Salute* was devised (Faggiano et al., 2010; Kreeft et al., 2009), this curriculum has a major focus on emotion recognition and management (> 25% of the overall program time) and does not include knowledge-based components because they were considered ineffective. The latter concern was confirmed by a formal mediation analysis (Giannotta, Vigna-Taglianti, Rosaria Galanti, Scatigna, & Faggiano, 2014) published after *Diario della Salute* was implemented. Additionally, the curriculum of *Diario della Salute* is relatively short compared to similar school-based programs implemented in Italy, which could have potentially resulted in considerable saving of resources. In this study, we assess the hypotheses that the *Diario della Salute* program increases subjective well-being and healthful behavior (reduction of smoking and alcohol drinking, and improvement in dietary habits and physical exercise) while reducing aggressive behavior.

## Methods

### Study Design

We conducted a cluster quasi-experimental study with two arms between January and May 2013 in two Northern Italian regions (Piedmont, Veneto) and in three Southern Italian regions (Apulia, Calabria, Sicily). The Ethics Committee of the Santa Croce e Carle Hospital of Cuneo, Italy, approved the study and we publicly registered it prior to its inception (Clinicaltrials.gov NCT01720199).

We asked each participating region to enroll at least four middle schools and 20 classes of 12- to 13-year-old students (on average, five classes per school). Schools participated in the study on a voluntary basis provided that they had a minimum of two classes of 12- to 13-year-old students, were state schools, and were not involved in other prevention programs. Eligible schools were given the choice of being in the intervention or in the comparison group, and underwent pairwise matching by number of students, an indicator of school-wide socioeconomic status, and geographical area to form two similar sets of schools. We assessed whether the distribution of the students’ sociodemographic characteristics and outcomes at baseline were balanced across groups. As the number of schools that chose the comparison group was greater than those that chose the intervention group, we discarded non-matching comparison schools. This procedure allowed us to retain all intervention schools while ensuring that the two sets of schools were as balanced as possible. Schools allocated to the intervention group implemented the full program, while those in the comparison group administered their regular curriculum and did not deliver any structured prevention activities that focused on the same outcomes as those that our program targeted.

### The Program

*Diario della Salute* [My Health Diary]^1^ is a school-based universal program designed to improve subjective well-being of youth aged 12–13 years old. It comprises (1) five standardized interactive units (each 2–4 h) on emotional, social and health issues administered by previously trained teachers (see Table 1); (2) a narrative booklet for teens that tells the story of four same-age students dealing with common developmental tasks such as identity formation, relationships with peers, conflicts with parents, and physical changes at puberty; and (3) a narrative booklet for parents that describes the experience of two families with teenage children that focuses on parent–child communication and their relationship during adolescence. Note that although there is no consensus on a single definition of subjective well-being, there is generally agreement that “at minimum, well-being includes the presence of positive emotions and moods (e.g., contentment, happiness), the absence of negative emotions (e.g., depression, anxiety), satisfaction with life, fulfillment and positive functioning” (Centers for Disease Control and Prevention, 2016).

**Table 1:** Program content, potential mediators and outcomes targeted

Program component	Activities	Materials	Duration (in hours)	Potential mediators targeted	Outcomes targeted
Unit 1: My emotions	Presentation, brainstorming, role-playing, recall of experiences associated to emotions, drawing, plenary discussion	Colored hats, marking pens, post-it notes, papers, posters, CD or MP3 player	3–4	Emotion recognition and management (Balluerka, Aritzeta, Gorostiaga, Gartzia, & Soroa, 2013; Schutte, Malouff, Simunek, McKenley, & Hollander, 2002)	Well-being
Unit 2: Beyond stereotypes	Presentation, group work, game, plenary discussion	Photos, cards, posters	2	Critical thinking (Werle, 2004)	Aggressive behavior
				Effective communication skills (Segrin, Hanzal, Donnerstein, Taylor, & Domschke, 2007)	Well-being
Unit 3: Becoming men & women	Presentation, role-playing, group work, homework, plenary discussion	CD or MP3 player, newspapers or magazines, posters	2–3	Critical thinking (Werle, 2004), interpersonal skills (Polan, Sieving, & McMorris, 2013)	Aggressive behavior
				Interpersonal skills (Siu & Shek, 2010)	Well-being
Unit 4: Exploring the world of adults	Presentation, group work, role-playing, feedback, plenary discussion	Cards	2	Critical thinking (World Health Organization, 2003), decision making (Stephens et al., 2009), peer-pressure resistance skills (Leung, Toumbourou, & Hemphill, 2014; Simons-Morton & Farhat, 2010)	Health behavior (smoking/alcohol)
Unit 5: Let’s keep fit	Presentation, feedback on aggregate analysis of individual worksheets, description of healthy and unhealthy behaviors, plenary discussion	Worksheets on individual diet and physical activity behavior filled out by each student for 1 week	3–4	Critical thinking (World Health Organization, 2003), self-efficacy (Dishman, 2004; Long & Stevens, 2004)	Health behavior (healthy eating, physical activity)

*Diario della Salute* was developed to address some of the limitations in school-based prevention and health promotion, building adolescents’ competence and including components designed to promote the psychological and behavioral well-being of the young. The program employs an interactive approach and focuses on the active engagement of children as generally is recommended (e.g., Cuijpers, 2002; Guerra & Bradshaw, 2008), and was designed after conducting a literature review of the potential mediators that would likely maximize the success of the program (Bonino, Cattelino, & Ciairano, 2005; Ferrer-Wreder, Stattin, Cass Lorente, Tubman, & Adamson, 2004; Jackson & Goossens, 2006; Roona, Streke, & Marshall, 2003).

This intervention followed a “train the trainer” model. Members of the research group delivered five two-day training courses (one course per study center) for 125 health professionals working in the field of prevention and health promotion. Health professionals trained middle school teachers in their area, selected the schools to enroll in the study, managed program implementation, and administered study surveys. Altogether, 191 teachers were trained by health professionals for 12.5 h over a maximum of four sessions and received a detailed program implementation manual. During their training sessions, teachers were made aware that *Diario della Salute* was designed in a modular fashion and were encouraged to tailor the program to the specific needs of their classes. Most of the trained teachers (*n *= 130) implemented the program during the second year of the Italian middle school to students aged 12–13. The number of teachers is greater than the number of classes in the intervention arm (*n *= 76) because in Italian middle schools there is usually more than one teacher per class. Sixty-one teachers did not implement the program, mainly because of simultaneous work commitments.

### Program Units

Table 1 presents a summary of the five interactive units implemented by trained teachers. Each unit’s aims and content were thoroughly discussed with teachers during the training phase, and teachers were provided with a comprehensive manual including detailed examples so that they could deliver the units using the correct format. In general, while program units were inspired by previous life skills programs, particularly *Unplugged*, their specific activities were original. A brief summary of each unit’s aim, duration and contents is provided herein.

Unit 1 ‘My emotions’ is planned to last 3–4 h and aims to improve the students’ capability to recognize and manage their own emotions, with the ultimate purpose to improve their well-being (Balluerka et al., 2013; Schutte et al., 2002). This unit relies on presentations, brainstorming, role-playing, drawing, and plenary discussions. For example, in one role-play activity students are asked to choose an emotion they wish to talk about (e.g., “happiness”) and then wear a colored hat representing that emotion (e.g., a blue hat). The teacher then asks questions to facilitate recollection of the circumstances during which the student experienced that particular emotion.

Unit 2 ‘Beyond stereotypes’ lasts 2 h and focuses on developing (1) critical thinking with the ultimate aim of reducing aggressive behavior (Werle, 2004), and (2) communication skills with the final purpose of improving well-being (Segrin et al., 2007). This unit is composed by several activities, including group work and team games. For instance, to prepare for one of these group-work activities, teachers would collect several photos representing persons in a way that does not reflect their real profession or role (for example, a rich businessman wearing cheap clothes). Students are asked to describe these photos and take guesses about the life of the persons depicted (e.g., where do they think they live, what do they do in the weekends, what kind of music they are into, are they popular, etc.). The teacher then compares students guesses with the real features of the persons involved, highlighting possible mismatches between appearance and reality.

Unit 3 ‘Becoming men and women’ is planned to last 2–3 h and targets critical thinking and interpersonal skills to reduce aggressive behavior and to improve well-being (Polan et al., 2013; Siu & Shek, 2010; Werle, 2004). This unit consists in several activities, including role-playing and group work. During the role-play activity, girls mimic how they think that boys behave during daily activities (e.g., tooth brushing, sports, when they meet someone they like, etc.) and boys do the same for girls. Teacher-led group-work follows to prompt reflection, for example by asking students what the similarities and differences were (for example, they could find out that many activities are performed more similarly than expected, whereas others are not).

Unit 4 ‘Exploring the world of adults’ lasts 2 h and targets potential mediators involved in smoking and alcohol behavior, such as critical thinking, decision making, and peer pressure resistance skills (Leung et al., 2014; Simons-Morton & Farhat, 2010; Stephens et al., 2009; World Health Organization, 2003). This unit relies mainly on role play and group work. For example, four students are asked to perform the beginning of a story during which the ‘cool kid’ asks the others to smoke a cigarette. Students are divided into small groups and each group is asked to imagine an ending for this story and perform it in front of the others. This is followed by teacher-led plenary discussion, during which the teacher encourages students to share their thoughts about how the four characters behaved and how they could have acted differently.

Unit 5 ‘Let’s keep fit’ is expected to last 3–4 h and aims to foster critical thinking and self-efficacy with the final purpose of favoring healthy eating and physical activity (Dishman, 2004; Long & Stevens, 2004; World Health Organization, 2003). Before the beginning of this unit’s activities, students are asked to fill out an anonymous worksheet to record their eating and physical-activity habits over 1 week. After about a week, the teacher involves students in a group work aimed at identifying their unhealthy behaviors and each group has to select three healthy behaviors and to decide to enforce them for a defined period (for instance, they might commit to use the stairs rather than the lift for at least 3 weeks).

### Data Collection

Prior to study commencement, trained health professionals informed parents and guardians about the study’s aims and methods and sought their active and written consent for student participation in the study. They also informed students that their participation was voluntary and they could opt out at any time. Health professionals also administered the questionnaires in classrooms, which required approximately 20 min per class. All questionnaires were anonymous, self-completed and identified by a self-generated code used to link pre- to post-intervention surveys (Galanti et al., 2007).

### Measures

#### Subjective Well-Being

Given that having multiple psychosomatic complaints is an indicator of little subjective well-being in preadolescence “as it reflects individual burden and personal experience related to negative life events in the social context of family, school and peers” (Inchley et al., 2016, p. 79), we measured subjective well-being using the WHO/Europe Health Behaviour in School-aged Children (HBSC) Symptom Checklist, a widely used instrument (Ravens-Sieberer et al., 2008). The HBSC Symptom Checklist has shown adequate psychometric properties in terms of reliability and validity in age groups similar to those included in the present study. The eight items of the HBSC Symptom Checklist ask about how often in the past 30 days the students suffered from headache, stomachache, backache, feeling low, irritability, nervousness, sleeping difficulties, and dizziness. Students could respond on a Likert scale by choosing one of the following answers: never (1), once or twice a month (2), once a week (3), more than once a week (4), and every day (5).

Since a positive emotional and social climate in school environment is predictive of both subjective well-being and better academic and social functioning (Roeser & Eccles, 1998; Van Ryzin, Gravely, & Roseth, 2009), we included three items in the study questionnaire to assess if students felt accepted by their classmates, got along with them, and were satisfied with their teachers: 4-point Likert scale with possible responses of (1) not at all, (2) a little, (3) quite, or (4) a lot.

#### Aggressive Behavior

We measured this behavior with 12-item extract of the Physical and Verbal Aggression Scale (Caprara & Pastorelli, 1993), a 20-item self-report scale that evaluates adolescent behavior intended to hurt others physically and verbally with items such as “I kick or punch” or “I said bad things about other kids” on a 4-point scale: never (1), sometimes (2), often (3), always (4). This scale was created originally in Italian and validated in several European countries (Caprara, Barbaranelli, Incatasciato, Pastorelli, & Rabasca, 1997) and the 12-item reduced version included in this study has been validated as part of the overall questionnaire validation study described further in this Methods section. We summed all items to provide a score of physical and verbal aggression for a maximum score of 48.

#### Health Behavior

Based on outcomes commonly used in the literature for similar prevention and health promotion programs (Faggiano et al., 2010; Sloboda et al., 2009), we specified the following past 30-d frequencies of health behavior: (1) cigarette smoking; (2) alcohol intoxication episodes (“how many times did you get drunk?”); (3) dietary habits (e.g., consumption of beverages and foods such as pop drinks, chips, vegetables, and fruits); and (4) moderate and heavy physical exercise (the latter as defined in the HBSC study as “any activity that increases your heart rate and makes you get out of breath some of the time for at least 60 min”; Cavallo et al., 2013, p. 48).

#### Sociodemographic Characteristics

We included age, sex, nationality, and a proxy measure of socioeconomic status in both descriptive and regression analyses. In surveys targeting adolescents, parental education is an acceptable proxy for socioeconomic status via family affluence (Galobardes, Shaw, Lawlor, Lynch, & Davey Smith, 2006). We used a binary variable to indicate socioeconomic status, coding high socioeconomic status as having at least one parent with a university degree or a high-school qualification, and coded as low socioeconomic status otherwise.

### Questionnaire Validation

We validated the evaluation questionnaire with a test–retest study of 49 students living in Piedmont, one of the five regions participating to the evaluation study. Demographic characteristics of these students were similar to those in the main evaluation study in terms of age at baseline (mean 12 years) and sex (53% boys). In test–retest comparisons, 55 questionnaire items (54%) were moderately correlated (0.40 ≤ Pearson’s *r* < 0.70), and 21 items (21%) were strongly or very strongly correlated (*r* ≥ 0.70). Cronbach’s alpha was 0.84, indicating good internal consistency.

### Sample Size

We conservatively anticipated a sample size of 2930 students based on the endpoint with the lowest expected baseline prevalence in the comparison group (π_1_), i.e. past 30-d smoking (Cavallo et al., 2013), while assuming 40% lower proportions in the intervention group (π_2_) based on the findings of a life-skills program implemented in Italy and assessed with a randomized trial (Gorini et al., 2014). We carried out sample size calculations assuming 5% type I error (z_α/2_) and 80% power (z_β_). We included an inflation factor (IF) of 1.9 to account for the cluster design. The IF was calculated assuming an intraclass correlation coefficient of 0.039 for past 30-d smoking, consistent with previous studies (Allara et al., 2015; Faggiano et al., 2010). We estimated the anticipated sample size using the following formula:

$${\text{Sample}}\;{\text{size}} = 2 \times \frac{{\left( {z_{\alpha /2} + z_{\beta } } \right)^{2} \times \left[ {\uppi_{1} \times \left( {100 -\uppi_{1} } \right) +\uppi_{1} \times \left( {100 -\uppi_{1} } \right)} \right] \times {\text{IF}}}}{{\left( {\uppi_{1} -\uppi_{2} } \right)^{2} }}$$

We carried out sample size calculations for all indicators measured in this study while leaving all other parameters unchanged.

### Attrition

**Fig. 1:**
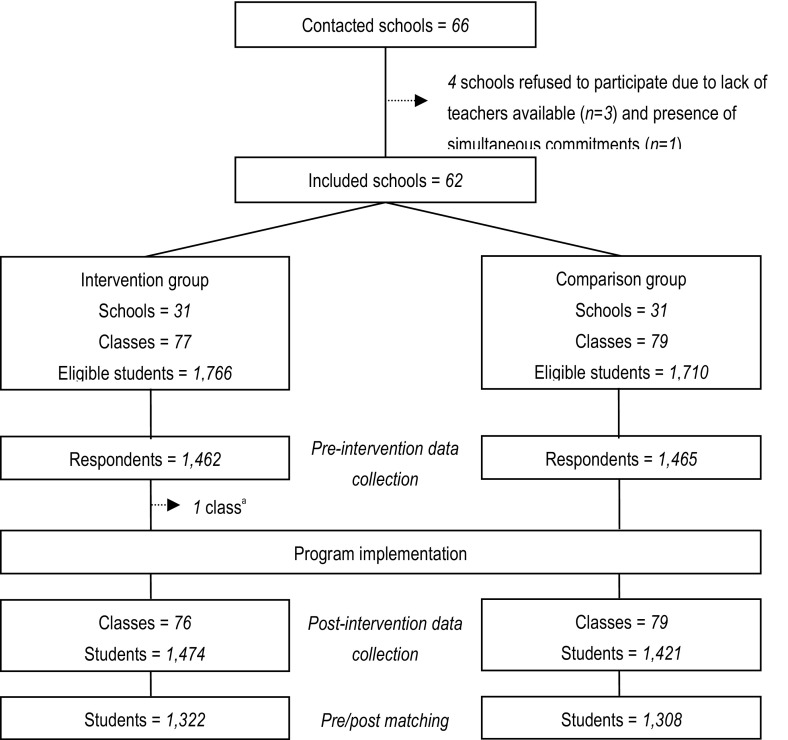
Study flowchart. ^a^One class dropped out after administration of the baseline survey

Out of the 66 middle schools contacted, four declined to participate due to lack of teachers available or presence of other simultaneous commitments (Fig. 1). The remaining 62 schools were distributed evenly between study arms. In the comparison group, 1462 out of 1766 eligible students (83%) filled out the baseline survey. In the intervention group 1465 out of 1710 eligible students (86%) filled out this survey. No school dropped out during the study. While no class dropped out before the baseline survey, one class in the intervention arm withdrew after the administration of the baseline survey and before the implementation of the program. None of the 79 classes allocated to the comparison arm dropped out. In the intervention group, 1322 students out of 1462 respondents (90%) completed both surveys. In the comparison group, 1308 students out of 1465 respondents (89%) completed both surveys.

### Statistical Analysis

We explored crude associations between study groups and outcome variables by performing cross tabulations of each outcome variable by study groups, both at baseline and after program implementation. We used Chi squared tests to compare proportions and *t* tests to compare means.

We used linear mixed-effects models to estimate adjusted program effects. Model 1 estimated the effect of the *Diario della Salute* program controlling for the outcome variable at baseline and assuming that the intercept varied randomly by school. This model can be written as η_ij_ = β_0_ + β_1_ × program_i_ + ∑_k_^l−2^ [β_k_ × baseline_i_ = k] + u_0j_, in which η_ij_ is a linear predictor whose link function is either logit for both binary and ordered categorical outcome variables, or identity for continuous outcome variables; program_i_ is a binary variable; baseline_i_ is either a binary (l = 2), categorical (l > 2) or continuous variable (by convenience, l = 2); u_0j_ is the random intercept, i.e., school, assumed to be normally distributed with mean zero and variance σ_u_^2^; and [] is an indicator function. Model 1 was nested within Model 2, which adjusted program effect for five potential confounders: outcome variable at baseline, sex, age, socioeconomic status, and nationality of parents. School-wide socioeconomic status was already accounted for by matching at the design stage.

## Results

### Baseline Sample Characteristics

Students’ demographic and socioeconomic characteristics were balanced across study groups (Table 2). Students had a mean age of 12 years (*SD* 0.54) and 50% were male. Approximately 45% had at least one parent with a university degree and 95% were of Italian descent.

**Table 2:** Baseline characteristics of the analysis sample

	Intervention	Comparison	Total	*p* ^a^
	*n *= 1322	*n *= 1308	*n *= 2630	
Mean age (*SD*)	12.1 (0.54)	12.1 (0.54)	12.1 (0.54)	0.784
Female gender (%)	674 (51.1)	636 (49.0)	1310 (50.1)	0.265
High socioeconomic status (%)^b^	472 (43.9)	510 (45.5)	982 (44.7)	0.443
Italian nationality (%)	1253 (95.0)	1250 (95.6)	2503 (95.3)	0.436
WHO/Europe HBSC Symptom Checklist (*SD*)	10.0 (3.56)	9.9 (3.66)	9.9 (3.60)	0.445
Classmate acceptance (*SD*)	3.3 (0.85)	3.3 (0.84)	3.3 (0.85)	0.344
Getting along with classmates (*SD*)	3.4 (0.76)	3.5 (0.76)	3.5 (0.76)	0.667
Satisfied with teachers (*SD*)	3.1 (0.83)	3.1 (0.83)	3.1 (0.83)	0.986
Past 30-day smoking (%)	30 (2.3)	63 (4.8)	93 (3.5)	< 0.001
Past 30-day alcohol intoxication (%)	33 (2.5)	37 (2.8)	70 (2.7)	0.860
Aggressive behavior (*SD*)	18.3 (4.30)	18.3 (4.41)	18.3 (4.25)	0.636

^a^Comparison tests between intervention and comparison arms. Chi squared tests for gender, socioeconomic status, nationality, smoking, and alcohol intoxication; *t* tests for the other variables

^b^High socioeconomic status indicates families in which at least one parent had a university degree or a high-school qualification. Low socioeconomic status indicated families in which at least one parent had a middle-school or elementary qualification

Outcome distribution at baseline appears generally balanced across study group, with the exception of past 30-d smoking, which was 2.3% in the intervention group vs 4.8% in the comparison group (*p *< 0.001).

### Implementation Fidelity

Almost three quarters of teachers in the intervention group implemented all of the program’s five units (*n *= 56 out of 76 classes = 74%), and others reported implementing less units (*n *= 4 four units = 5%, *n *= 10 three units = 13%, *n *= 5 two units = 7% and *n *= 1 one unit = 1%). Unit one was the most popular, implemented in 75 classes (99%), followed by unit two (97%), unit three (88%), unit four (82%), and unit five (78%). Although we did not require units to be delivered in a particular order and teachers were free to tailor the program to the needs of their class, the first units were those most closely related to the program main objective of improving well-being. Teachers reported on the lack of dedicated time and space as the main barrier to program implementation and prioritized the units deemed most appropriate to the needs of their students.

With regard to the level of students’ participation in program-related classroom activities, 98% of the teachers stated that the level of participation was high or rather high. Eighty-nine percent of teachers stated that the implementation of classroom activities was easy or rather easy.

### Program Effect on Subjective Well-Being

When controlling for pre-intervention levels of the outcome, age, sex, SES, and nationality of parents, students in the intervention group had a 0.38 greater mean score of the WHO/Europe HBSC Symptom Checklist (95% confidence interval 0.14–0.66) in the previous 30 d than did students not exposed to the intervention (Table 3), reflecting reduced subjective well-being.

**Table 3:** Subjective well-being

Outcome	Pre-intervention		Post-intervention		Mean difference (95%CI) (Model 1)^a^	Mean difference (95%CI) (Model 2)^b^
	Intervention	Comparison	Intervention	Comparison		
	*n *= 1322	*n *= 1308	*n *= 1322	*n *= 1308		
WHO/Europe HBSC Symptom Checklist Score (*SD*)	10.0 (3.56)	9.9 (3.66)	10.5 (3.81)	10.0 (9.74)	0.38**	0.38**
% Missing	3.8	5.0	2.0	2.0	**(0.16–0.60)**	**(0.14–0.66)**
Classmate acceptance *(SD*)	3.3 (0.85)	3.3 (0.84)	3.2 (0.87)	3.3 (0.82)	− 0.04	− 0.03
% Missing	0.7	0.7	0.4	0.5	(− 0.10–0.01)	(− 0.10–0.03)
Getting along with classmates (*SD*)	3.4 (0.76)	3.5 (0.76)	3.3 (0.81)	3.4 (0.79)	− 0.03	− 0.04
% Missing	0.5	0.8	0.4	0.4	(− 0.08–0.02)	(− 0.09–0.02)
Satisfied with teachers (*SD*)	3.1 (0.83)	3.1 (0.83)	3.1 (0.87)	3.1 (0.83)	− 0.002	− 0.04
% Missing	0.5	0.9	0.5	0.4	(− 0.08–0.08)	(− 0.12–0.05)

Bold values indicate estimates for which there is evidence of an effect at the 5% level

*HBSC* Health Behaviour in School-Aged Children

^a^Model 1: program effect adjusted for outcome at baseline

^b^Model 2: program effect adjusted for outcome at baseline, socioeconomic status, continuous age, sex, nationality of parents

### Program Effect on Health Behavior and Aggressive Behavior

There was no evidence of program effect on smoking, alcohol drinking, and aggressive behavior (Table 4), diet or emotional eating, physical activity or sedentary lifestyle (tables not shown, available from the first author upon request).

**Table 4:** Smoking, alcohol drinking and aggressive behavior

Outcome	Pre-intervention		Post-intervention		Odds ratio (95% CI) (Model 1)^a^	Odds ratio (95% CI) (Model 2)^b^
	Intervention	Comparison	Intervention	Comparison		
	*n *= 1322	*n *= 1308	*n *= 1322	*n *= 1308		
*A. Categorical outcomes*						
Past 30-d smoking (%)						
No	96.8	94.9	94.9	94.4	1.09 (0.64–1.85)	1.15 (0.70–1.89)
Yes	2.3	4.8	4.6	5.5		
Missing	1.0	0.3	0.5	0.1		
Past 30-d alcohol drinking (times) (%)						
No	79.7	77.3	78.9	75.9	0.79 (0.58–1.08)	0.80 (0.58–1.09)
1–2	12.0	12.8	11.9	13.4		
3 +	7.6	8.8	8.3	9.6		
Missing	0.8	1.2	0.9	1.2		
Past 30-d alcohol intoxication (%)						
No	96.3	96.0	96.0	96.5	1.37 (0.84–2.24)	1.22 (0.70–2.13)
Yes	2.5	2.8	3.3	2.5		
Missing	1.2	1.2	0.8	1.0		
*B. Continuous outcomes*						
Aggressive behavior (*SD*)	18.3 (4.30)	18.3 (4.41)	18.8 (4.40)	18.7 (4.62)	− 0.06 (− 0.41–0.29)	0.03 (− 0.33–0.39)
% Missing	1.7	1.7	1.3	1.3		

^a^Model 1: program effect adjusted for outcome at baseline

^b^Model 2: program effect adjusted for outcome at baseline, socioeconomic status, continuous age, sex, nationality of parents

## Discussion

The quasi-experimental evaluation presented in this paper involved 2630 preadolescents in five socioeconomically and demographically diverse Italian regions. The *Diario della Salute* [My Health Diary] program was associated with students reporting increased somatic and psychological health complaints which indicated a reduced subjective well-being compared to those not in the program. This finding is quite isolated in the literature due to the paucity of studies that have measured the direct effect of health promotion programs on well-being, as opposed to the abundance of studies that have focused on health-related behaviors such as physical activity and smoking (Langford et al., 2014). However, the literature provides some evidence that can serve to explain the results of our study. Although adolescence is traditionally seen as a life period characterized by relatively good health, most adolescents experience some level of physical or emotional distress and are susceptible to psychosomatic complaints (Hetland, Torbjorn, & Aaro, 2002). A common view in psychology is that somatic complaints develop as a result of the psychological reinforcement of physiological signals (Eriksen & Ursin, 2004). By directing attention inwards and giving more attention to signals from the body, an increased awareness of pain and suffering may occur. The increase in somatic and psychological health complaints could be interpreted as a possible iatrogenic effect of the program, related to the structure or content of the program. However it is also possible that students in the intervention group acquired a greater ability to recognize their own emotions, which is key ingredient of socioemotional competence and positive psychosocial adjustment (Adams & Berzonsky, 2003). This awareness, while beneficial, however, may have led to reduced well-being in this study. Future studies assessing the effectiveness of interventions to promote well-being might want to differentiate between these two possible interpretations by studying more closely how interventions influence emotion recognition and awareness skills.

The program did not appear to have effects on either physical health or aggressive behavior. Although it is possible that the program did not address these outcomes with sufficient intensity as they were not its principal focus, it may also be that the short follow-up period of this study (1 month after completing program implementation) was not sufficient to capture changes in these areas. The strict deadline enforced by the project funder for the completion of the study did not allow carrying out a further follow-up survey at a later time to detect later-onset program effects which are uncommon but possible.

The main limitation of the study concerns the lack of randomized allocation to study groups. In Italy, the evaluation of prevention programs is uncommon, randomized controlled trials are rare (Coffano, 2009), and schools are resistant to randomization procedures. In general, we believe that the schools that chose the intervention group did so either because of a greater perceived need (especially those located in the study’s most deprived areas) or because of greater interest in the program (especially in schools located in the most affluent areas). Since schools did not receive any compensation for participating in this study, this factor would have not influenced schools’ choice of their study group. We did not find evidence of a difference between study groups by sociodemographic characteristics and outcomes at baseline, which suggests a low risk for selection bias at least in regards to the variables examined. Further, our statistical analyses reduced the potential for confounding by accounting for pre-intervention outcome levels and sociodemographic characteristics. Another potential limitation concerns the program’s lack of direct assessment of implementation fidelity. Given our purpose to evaluate the effects of the program in a real-life setting, we relied only on self-report information obtained from the teachers with a pencil-and-paper questionnaire that teachers filled out after program implementation. However, the program was manualized in detail, all teachers received the same training, and all students and parents received the same booklets as appropriate. One program feature that could have influenced the overall program effect was the freedom given to teachers to tailor the program to the needs of their class. In post hoc adjusted analyses we observed strong evidence (*p *= 0.001) of a positive association between number of units implemented and the WHO/Europe Symptom Checklist instrument (0.08 linear increase in score per each unit implemented, 95% CI 0.04–0.14), reflecting worse subjective well-being in a linear dose–response fashion. This suggests that although the freedom to tailor the program to the class needs is likely to result in different program effects, even the minimum implementation of the program (one unit) was sufficient to result in strong evidence of an effect on the main outcome. Although it is possible that heterogeneity in program implementation (due to the flexibility of the program, one of its main strengths) may have influenced the size of the overall program effect, it is unlikely to have modified the overall interpretation of this study’s findings.

At least three key strengths may be noted for this evaluation study. First, because we conservatively based our sample size estimations on the least prevalent indicator (i.e., smoking behavior) the study was sufficiently powered to detect changes in subjective well-being, which was the principal focus of the program, and adequately powered to capture changes in the other objectives. Second, our evaluation was conducted in real-life settings. A large part of the literature comprises efficacy studies of programs carried out in controlled research settings (Faggiano et al., 2014), and it is unclear to what extent they may be generalizable for practice. Since *Diario della Salute* was evaluated under real-life (that is, effectiveness) conditions, the generalizability of our findings is maximized. Third, our study was conducted in five geographically and socioeconomically diverse Italian administrative regions, which increases the external validity of our findings. Our findings may also serve to show that the development and large-scale evaluation of a structured preventive program is feasible even in low-resource settings, provided that local health authorities and the education system and administrators are committed to the project.

In conclusion, our large multicenter evaluation study of *Diario della Salute* yielded an unexpected finding of reduced well-being as a result of program exposure, based on an increased perception of psychosomatic complaints among preadolescents. This could be explained by either an iatrogenic effect or an increase in emotional recognition skills that are positively associated with well-being over the lifespan. While higher emotional competence is associated with greater well-being, the program in its present form should not be disseminated due to the possibility of adverse unintended effects.

## Footnotes

1 http://www.diariodellasalute.it.
